# THE CARE RECIPIENT’S PERSPECTIVE ON QUALITY OF CARE: DIFFERENT APPROACHES IN NURSING HOMES

**DOI:** 10.1093/geroni/igz038.283

**Published:** 2019-11-08

**Authors:** Hilde Verbeek, Kimberly Van Haitsma

**Affiliations:** 1 Department of Health Services Research, Care and Public Health Research Institute (CAPHRI), Faculty of Health, Medicine and Life Sciences,, Maastricht, Netherlands; 2 Penn State, University Park, Pennsylvania, United States

## Abstract

In long-term care, there has been an ongoing shift focused on person-centered care, positioning the care recipient at the core of good quality of care. This has resulted in more emphasis on care recipients’ preferences and experiences with the care they receive. In the Netherlands, Germany and the United States the need to focus on and assess quality from the care recipient’s perspective has emerged. This symposium presents four different approaches to quality of care from the care recipient’s perspective. The first speaker will focus on a narrative approach to assess experienced quality of care in nursing homes. The second speaker will present an observational method to assess the level of autonomy provided to people with dementia in nursing homes. The third speaker will present findings about the use of specialist health care in nursing homes as an indicator for quality of care. The last presentation will address the importance of preferences in quality of care. Defining, assessing and improving experienced quality of care from the care recipient’s perspective is an ongoing challenge, as each care recipient’s preferences and needs differ. It is important to assess in order to monitor that care is being tailored to the care recipient and to identify possible interventions that can enhance experienced quality of care.

